# Functional Adaptation and Emergent User Solutions in Domestic Tasks: Supporting Aging in Place Through a Field Study on Design Challenges Among Older Adults in Chile

**DOI:** 10.3390/healthcare13121369

**Published:** 2025-06-07

**Authors:** Juan Carlos Briede Westermeyer, Leonardo Madariaga Bravo, Eduardo Piñones, Karina Neira-Zambrano, Natalia Debeluck Plentz, Cristhian Pérez-Villalobos

**Affiliations:** 1 Department of Engineering Design, Universidad Técnica Federico Santa María, Avda. España 1680, Valparaíso 2390123, Chile; leonardo.madariaga@usm.cl (L.M.B.); eduardo.pinones@usm.cl (E.P.); natalia.debeluck@usm.cl (N.D.P.); 2 Department of Design and Theory of Architecture, Faculty of Architecture, Construction and Design, Universidad del Bío-Bío, Avda. Collao 1202, Concepción 4051381, Chile; kneira@ubiobio.cl; 3 Medical Education Department, School of Medicine, Universidad de Concepción, Concepción 4070386, Chile; cperezv@udec.cl

**Keywords:** aging in place, functional adaptation, emergent user solutions, domestic routines, inclusive product design

## Abstract

Maintaining quality of life through functional autonomy is crucial for supporting aging in place. While assistive technologies and architectural adaptations have received significant attention, there is limited knowledge on how older adults independently adapt domestic routines using everyday household products. **Background/Objectives**: This study aimed to explore how functionally independent older adults manage key domestic tasks and to identify user-driven adaptations that could inform inclusive product design. **Methods**: We conducted a qualitative field study involving non-participant observations and in-depth case studies with 20 older adults aged 65–85 living in urban Chile. Participants were observed while performing cooking, dishwashing, and waste disposal activities. Thematic analysis and axial coding, based on grounded theory principles, were applied to identify adaptation strategies and usability barriers. **Results**: Participants employed a range of adaptation strategies across tasks, including temporal redistribution of effort, spatial reorganization, informal tool use, and reliance on social support. These adaptations reflected creative and situated responses to physical and environmental constraints. Many strategies could be interpreted as emergent user solutions, offering practical insights for the inclusive and low-cost redesign of everyday objects. **Conclusions**: Older adults actively modify their interactions with domestic environments to preserve autonomy and functionality. Recognizing and incorporating these emergent user adaptations into product design processes can strengthen inclusive design practices, support aging in place, and inform public health strategies aimed at promoting independence among aging populations.

## 1. Introduction

Population aging is a growing global phenomenon that poses significant challenges to healthcare systems, the built environment, and the quality of life of older adults. In Chile, it is projected that more than 30% of the population will be over the age of 60 by 2050, highlighting the need to promote functional autonomy and support aging in place [1]. Aging in place refers to the ability of older individuals to continue living independently and safely in their own homes, without requiring institutionalization [2,3].

According to the World Health Organization, healthy aging involves not only the absence of disease but also the capacity to carry out meaningful activities and actively engage with one’s environment [1]. In this context, instrumental activities of daily living (IADLs)—such as cooking, dishwashing, and waste disposal—are critical for maintaining independence and ensuring well-being in old age [4].

Although significant research has been dedicated to assistive technologies [5,6,7,8] and architectural interventions [9,10], much less is known about how older adults adapt their everyday domestic practices using ordinary household objects. These domestic routines involve physical, cognitive, and emotional demands and are often reshaped by users in ways that remain invisible to mainstream design and policy. In Latin American urban settings, there is a notable lack of qualitative research that explores how older adults maintain autonomy through spontaneous and personalized adaptations—especially when access to specialized products is limited. While assistive technologies are commonly associated with devices such as grab bars, walkers, or smart monitoring systems [11,12], this study adopts a broader interpretation that includes informal, low-tech, and repurposed objects that older adults use to maintain autonomy. These everyday adaptations—such as modified tools, supportive furniture arrangements, or creative use of utensils—can function as assistive technologies in practice, even if they are not formally recognized as such. This perspective expands the traditional scope of assistive design by considering the lived experience and resourcefulness of users.

In this context, instrumental activities of daily living (IADLs)—such as cooking, dishwashing, and waste disposal—are critical for maintaining independence and ensuring well-being in old age [4]. These domestic routines involve physical, cognitive, and emotional demands and are deeply shaped by the usability and accessibility of household environments.

Although significant research has been dedicated to assistive technologies [5,6,7,8] and architectural interventions [9,10], much less is known about how older adults adapt their everyday domestic practices using ordinary household objects. Studies conducted in Latin American contexts have identified numerous environmental barriers within older adults’ homes that compromise daily functioning and increase the risk of accidents. For example, ref. [13] found that sensory, motor, and cognitive limitations often interact with inadequate domestic layouts—such as poor lighting, slippery surfaces, or inappropriate furniture—hindering autonomy and safety. Similarly, ref. [14] highlights how age-related frailty and physical decline are exacerbated by built environments that do not accommodate older adults’ needs, reinforcing the importance of accessible and adaptive design in maintaining quality of life.

Despite increasing attention to inclusive design and smart technologies for aging in place [15,16] there is a notable gap in understanding how older adults independently adapt their domestic routines using the tools and environments already available to them. Most studies focus on externally provided solutions, while overlooking the emergent, user-driven adaptations that take place in ordinary settings—particularly in low-resource, urban environments. As ref. [17] demonstrates, older persons with chronic conditions actively strive to maintain autonomy through creative and situational strategies that negotiate their limitations and surroundings. Similarly, ref. [18] shows that older adults employ a range of compensatory techniques—including temporal adjustments, tool improvisation, and reliance on social support—to overcome everyday challenges. Furthermore, ref. [19] emphasizes the potential of mapping spatial and temporal patterns of domestic routines to inform inclusive and context-sensitive design responses.

In response to these challenges, user-centered and co-design approaches have emerged as effective strategies for developing socially acceptable and sustainable solutions that respond not only to physical limitations but also to emotional and symbolic needs [20,21,22,23,24].

These findings underscore the importance of recognizing emergent user solutions as valuable foundations for inclusive, low-cost product redesigns aligned with the capabilities and contexts of aging users. However, further empirical research is needed to explore how functionally independent older adults manage domestic tasks in real-life settings and what design insights can be drawn from their situated problem-solving behaviors.

In addition to design-centered approaches, this study draws conceptually from gerontological models that understand aging as a dynamic process of adaptation between individuals and their environments. The ecological model of aging [25] and subsequent frameworks emphasize the importance of the person–environment fit, functional competence, and behavioral adaptation as individuals face age-related challenges. These models provide a useful lens for interpreting the compensatory strategies observed, which reflect efforts to maintain autonomy through interaction with both physical and symbolic dimensions of the domestic space.

*Research Questions:*

How do functionally independent older adults adapt and manage domestic tasks such as cooking, dishwashing, and waste disposal in their homes, and what design insights can be drawn from their everyday compensatory strategies?

To address these questions, this study conducts a situated qualitative exploration of domestic routines among older adults in southern Chile. It seeks to generate empirical and conceptual contributions that support aging in place by informing inclusive and context-sensitive product design [26,27,28] aimed at preserving autonomy and improving quality of life in later life. Empirically, this study offers detailed, situated observations of how older adults in Chile negotiate common household tasks, a perspective underrepresented in the global aging literature. Conceptually, it contributes to the understanding of functional adaptation as a dynamic process involving improvisation, bodily awareness, and interaction with the material environment. These findings enrich existing models of aging in place and user-centered design by highlighting the symbolic and creative dimensions of domestic autonomy.

The article is structured as follows. Section 2 presents the methodological approach, detailing the qualitative design, participant selection, and data collection techniques used in field study. Section 3 outlines the main findings, focusing on the strategies older adults employ to adapt to domestic routines and the contextual factors that influence these adaptations. Section 4 discusses the implications of these findings for inclusive product design, relating them to the existing literature and conceptual frameworks such as compensatory behaviors and user-driven innovation. Finally, Section 5 offers conclusions and recommendations for design practice, aging policy, and future research directions.

## 2. Materials and Methods

### 2.1. Study Design

**Figure 1:**
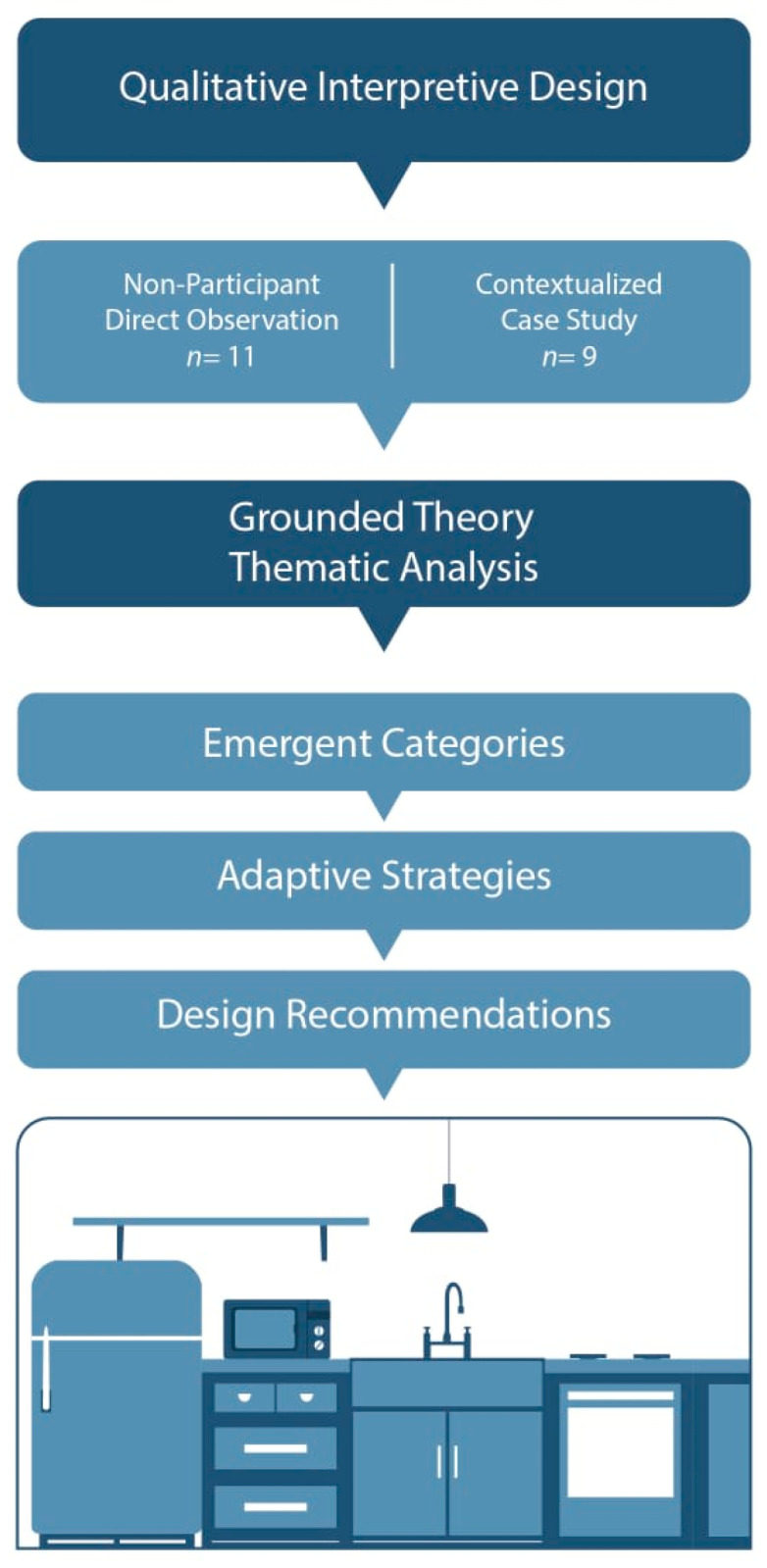
Methodological structure, from qualitative design to data collection and grounded theory analysis.

This study used a qualitative, exploratory, and interpretative design aimed at understanding how functionally independent older adults in urban settings perform three key domestic activities: cooking, dishwashing, and waste disposal. A combination of non-participant observation and grounded theory-based analysis was used to document everyday practices, adaptive strategies, and functional interactions with household objects. Grounded theory is a qualitative methodology that allows theory to emerge from real-world data through systematic collection and analysis (Figure 1) [29].

### 2.2. Participants and Sampling

The sample consisted of 20 functionally independent older adults, aged 65 to 85, residing in private homes located in urban and peri-urban areas of the Biobío Region, southern Chile. Participants were selected using purposive sampling [30], based on prior data collected through a survey of 399 older adults conducted as part of the Fondecyt Regular Project No. 1171037—a publicly funded research initiative supported by the National Agency for Research and Development (ANID) in Chile.

All participants self-identified as functionally independent and were classified as such by community health records. Initial screening was conducted using a structured questionnaire developed as part of the same Fondecyt project. This tool included self-rated ADL/IADL performance and task difficulty, enabling the selection of individuals who reported moderate difficulty but no dependency in key domestic activities.

Recruitment was carried out through community organizations and local networks. All participants provided written informed consent. The study protocol, including all instruments and procedures, received approval from the Institutional Ethics Committee (Ethics Report No.: 263/2020).

The decision to include 20 participants was based on two criteria: (1) theoretical saturation, in line with grounded theory principles, as no new conceptual categories or significant variations were identified after the 18th participant; and (2) the observed diversity in domestic settings and adaptive strategies, which provided a rich representation of functional experiences. This sample size is consistent with other qualitative studies focused on daily living activities among older adults, which prioritize contextual depth over statistical generalizability [29,31].

While the sample size of 20 participants may appear limited, it aligns with qualitative research standards that prioritize depth and contextual understanding over statistical generalization. The sample was purposefully constructed to capture a range of functional experiences, and data collection continued until theoretical saturation was reached. The recent literature confirms that smaller sample sizes are adequate when they provide rich, relevant insights and fulfill methodological principles such as saturation and heterogeneity [32]. Moreover, similar qualitative studies involving older adults have drawn meaningful conclusions from comparably small samples when demographic diversity and in-depth data collection have been ensured [33].

To enhance the transferability of our findings, we provide additional sociodemographic details of the participant sample (n = 20). The group included 11 women and 9 men, with an age range of 60 to 85 years (mean ≈ 72). Living arrangements varied: seven participants lived alone, five with partners, and eight in multigenerational households. Most belonged to low- to middle-income backgrounds and were retired, with occupations such as a seamstress, caregiver, or teacher reported. Common health conditions included arthritis, hypertension, and visual impairments, although some participants reported no chronic illness. All participants resided in urban or peri-urban areas of central Chile, including Viña del Mar, Valparaíso, and Villa Alemana. This diversity of profiles provides a rich context for exploring real-life functional adaptations in domestic environments.

The selection of cooking, dishwashing, and waste disposal as focal activities was based on a preliminary survey of 399 older adults, in which these tasks were identified as the most frequent and physically or environmentally challenging. This choice is further supported by recent studies that emphasize the significance of these IADLs in maintaining health and safety among older adults. For example, ref. [34] found a strong association between limitations in IADLs and poorer self-rated health among community-dwelling older adults. Ref. [35] highlighted the physical difficulties and safety concerns faced by older people when performing cooking tasks, while ref. [36] explored the use of smart-home technologies to monitor IADLs—including meal preparation and waste management—among older adults with cognitive impairment. The selection of cooking, dishwashing, and waste disposal as the focal tasks was grounded in the aforementioned survey results. These three IADLs emerged as the most frequently performed and most commonly reported as challenging. In addition to their physical demands—requiring manual dexterity, posture control, and mobility—they also carry symbolic and emotional significance. As such, they provide a multidimensional lens through which to examine functional adaptation strategies in everyday domestic life.

### 2.3. Data Collection Techniques

Two complementary strategies were used:

- Non-participant direct observation (n = 11): Real-time observations of domestic tasks were conducted in participants’ homes. A structured protocol was used, including photographic records, a chronological description of the phases of each activity, and notes on tools used, body movements, and identified barriers. This technique allows researchers to document behaviors in context without intervening in the activity [31]. The observation sheet included predefined fields to record object use, spatial limitations, and body strategies.
- In-depth case studies (n = 9): A context-sensitive observation guide was used, accompanied by field notes, spatial sketches, and documentation of informal adaptations. This method helped capture the symbolic and emotional dimensions of domestic routines [37].

In both strategies, participants were observed while performing at least one of the following activities: cooking (n = 4), dishwashing (n = 3), and taking out the trash (n = 4).

**Figure 2:**
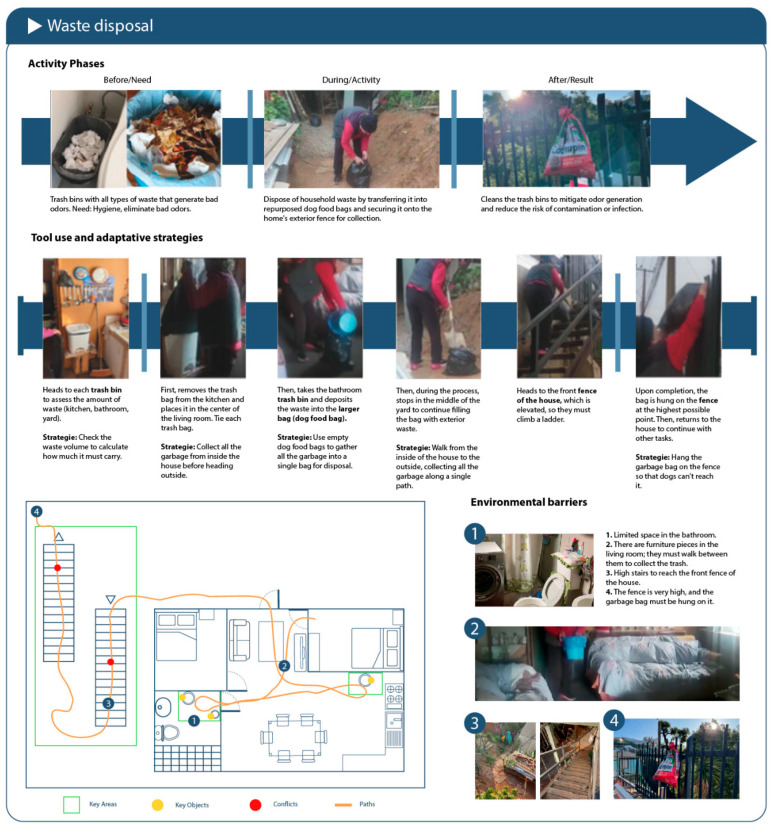
Example of the observation sheet used to document activity phases, tool use, adaptive strategies, and environmental barriers.

This methodological approach aligns with user-centered design principles and supports the research aim of identifying real-world compensatory strategies that older adults use in their homes, generating design-relevant insights grounded in observed behavior (Figure 2).

### 2.4. Data Analysis

The data was analyzed using thematic analysis and axial coding according to grounded theory principles [38]. The process included

- Open reading and initial coding [39];
- Grouping of categories around central phenomena;
- Identification of causal conditions, consequences, and action/interaction strategies.

The analysis was supported by ATLAS.ti v23 software, which provides advanced tools for qualitative data analysis. Triangulation among researchers and theoretical saturation were used to ensure the reliability of the analytic process [40].

The coding process began with in vivo coding of participant actions and comments (e.g., “I place the dishes in order to avoid bending” or “I wash everything at once to get it over with”), which evolved into descriptive codes such as “sequencing strategies” or “effort minimization”. These were later grouped into axial categories including “temporal redistribution”, “ergonomic adaptation”, and “symbolic meaning of routines”.

**Figure 3:**
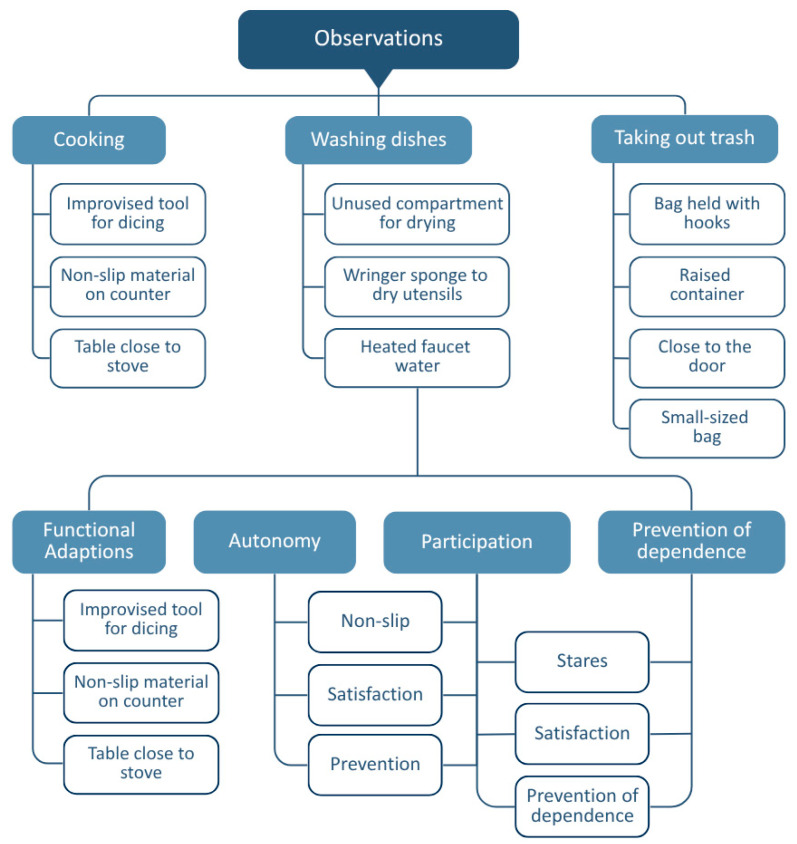
Coding tree.

A coding tree (Figure 3) was developed to visualize the progression from initial codes to axial categories, supporting analytical transparency and trustworthiness as recommended in thematic analysis guidelines [41].

Additionally, Table 1 presents illustrative quotes corresponding to each major theme, highlighting the participants’ voices and grounding analytical claims in the field data.

Although formal member checking was not implemented due to logistical constraints, results were validated through regular team coding sessions, analyst triangulation, and alignment with previous findings from the larger Fondecyt study. Future iterations may incorporate participant feedback in the design of assistive strategies, as part of a follow-up co-design phase.

### 2.5. Ethical Considerations and Data Availability

This study followed the ethical principles of the Declaration of Helsinki. Anonymized data, non-identifiable contextual images, observation protocols, and instruments are available upon reasonable request to the corresponding author. No automated algorithms or proprietary software beyond ATLAS.ti were used. There are no access restrictions on the materials employed.

Data was analyzed using thematic coding with an abductive logic, combining inductive insights from field observations with sensitizing concepts drawn from the aging and design literature. During the iterative process of analysis, three overarching analytical categories were defined to structure the findings:

1. Common Phases refer to recurring temporal segments or stages that organize how older adults approach domestic tasks (e.g., preparation, execution, and closure). These phases were used to understand the procedural logic of everyday routines.
2. Key Elements describe the material, spatial, and relational components that are critical to task execution and adaptation—such as specific tools, body postures, surfaces, or interactions with others.
3. Contextual Variations account for differences across participants due to personal characteristics, household configurations, or socio-spatial conditions and highlight how adaptations are shaped by local contingencies.

These categories guided the organization of the results and enabled a coherent comparison across cases, while preserving the situated nature of each participant’s routine.

The potential influence of the Hawthorne Effect was considered in our research design and interpretation. While it is recognized that participants may modify their behavior when being observed, we sought to minimize this effect by conducting observations in familiar home settings and allowing participants to proceed at their usual pace, without task prompting or interference. Additionally, prolonged immersion in some cases helped reduce reactivity. Importantly, many of the adaptive behaviors documented (e.g., use of informal tools, resting strategies, or skipping steps) were consistent with the participants’ reported routines and were not idealized or socially desirable performances, suggesting the ecological validity of the observations [42].

This study followed the principles of qualitative rigor and transparency outlined in the COREQ (Consolidated Criteria for Reporting Qualitative Research) checklist [43]. Key components addressed include the researcher characteristics and reflexivity; sampling strategy and setting; data collection methods; analytical approach (including coding and triangulation); and presentation of quotations to support interpretations. A completed COREQ checklist is included as Supplementary File S1 to guide the evaluation of this manuscript’s methodological reporting.

## 3. Results

### 3.1. Cooking: Preliminary Findings

Cooking emerged as a complex domestic activity among older adults, involving a detailed sequence of actions with significant physical, emotional, and symbolic engagement. Based on observations in four homes (SETs 3, 5, 18, and 20), the task was analyzed in terms of its phases, needs, adaptations, and common challenges.

#### 3.1.1. Common Phases Identified

- Planning and organization: preparing ingredients, cleaning surfaces, and arranging utensils.
- Preparation: washing, peeling, chopping, mixing—techniques varied depending on the dish (e.g., sautéing, boiling, kneading).
- Cooking and monitoring: managing timing using clocks or intuition, stirring, and handling heat sources.
- Cleanup and closure: washing dishes, storing leftovers, and cleaning surfaces.

#### 3.1.2. Key Elements

- Structured planning: Some participants anticipated steps and even prepared ingredients a day in advance (e.g., soaking beans), reducing both cognitive and physical demands.
- Informal adaptations and support: Pressure cookers, timers, and help from family members were common in physically demanding tasks such as cutting squash.
- Physical strain and risks: Cooking involved standing, walking, bending, lifting, and repetitive hand movements, contributing to fatigue and the fall risk.
- Symbolic and emotional dimension: Cooking was not only a functional task but also an act of care and continuity, linked to personal and cultural identity.

#### 3.1.3. Contextual Differences

**Figure 4:**
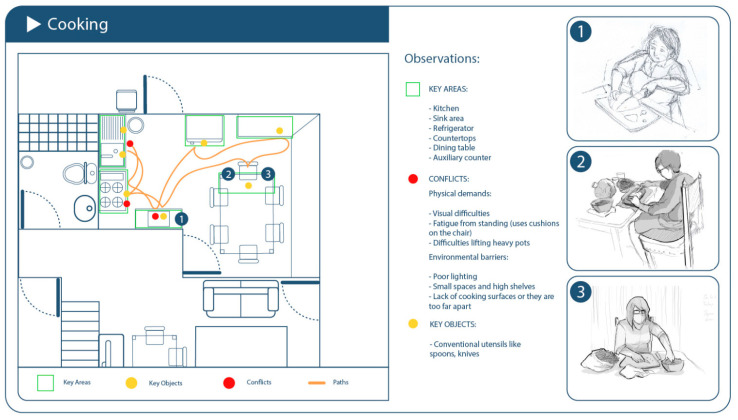
Schematic of the domestic environment observed during the cooking activity.

- Most participants used traditional kitchen layouts, but small spaces and high shelves created ergonomic barriers.
- Conventional utensils were dominant; ergonomic tools were rare or improvised (e.g., long-handled wooden spoons) (Figure 4).

### 3.2. Dishwashing: Preliminary Findings

Although seemingly simple, dishwashing involved a cognitively and physically sequenced process. Observations in three homes (SETs 4, 10, and 13) revealed routines that emphasized order, energy management, and cleanliness.

#### 3.2.1. Common Phases

- Collecting and organizing dirty dishes.
- Washing with sponges or steel wool.
- Rinsing and placing items in a drying rack.
- Drying and storing clean items.
- Cleaning the workspace.

#### 3.2.2. Key Elements

- Sequential structure: Participants tended to wash less greasy items first and left the pots and pans for last, thereby optimizing effort and water use.
- Accessible resources and spontaneous adaptation: Traditional tools were used with minimal assistive technologies. Some reused water or used it for watering plants, reflecting environmental awareness.
- Physical demands and risks: Prolonged standing, repetitive arm motions, and slippery surfaces contributed to fatigue and posed fall risks.
- Symbolic value of order and care: Maintaining a clean kitchen fostered a sense of control and dignity, reinforcing independence.

#### 3.2.3. Contextual Differences

**Figure 5:**
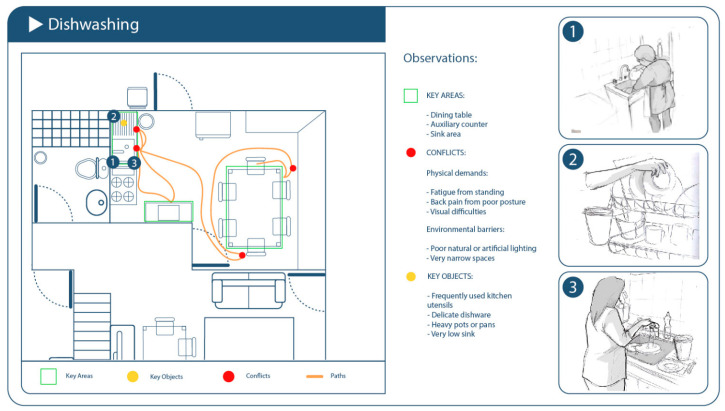
Schematic of the domestic environment observed during the dishwashing activity. It highlights frequently used areas, movement paths, improvised support points, and spatial conditions influencing ergonomics and perceived physical effort.

- Availability and usability of hot water systems varied. Some participants needed to manually ignite gas water heaters (calefonts) located outside the kitchen or on patios, which introduced delays, physical strain, and safety concerns—especially during colder months or for those with reduced mobility. In contrast, others had automated or centrally located systems that facilitated seamless access to hot water during dishwashing.
- Ergonomic limitations due to sink and counter dimensions were common. Households with small sinks or limited counter space reported greater physical effort and awkward postures when washing and rinsing dishes. These spatial constraints led to adaptations such as using additional basins, shifting tasks to other surfaces, or reducing washing frequency (Figure 5).

### 3.3. Waste Disposal: Preliminary Findings

#### 3.3.1. Common Phases

Participants typically followed a consistent three-phase sequence in waste disposal: accumulation, transport, and elimination. The accumulation phase involved collecting waste in localized containers (e.g., kitchen bins or bags) until full. The transport phase required carrying the container or bag to a disposal point, which often involved navigating through the home or building. The elimination phase consisted of emptying the waste into external containers or communal collection points, and occasionally cleaning or replacing the container.

#### 3.3.2. Key Elements

Key elements in this routine included the physical characteristics of waste containers (size, shape, lid mechanism), the path to disposal (stairs, thresholds, elevator access), and the interaction with community infrastructure (type of waste bin, timing of collection). Participants also relied on tools such as walking supports or bags with handles. Emotional discomfort linked to odors, hygiene, or perceived indignity of the task was mentioned by some participants.

#### 3.3.3. Contextual Variations

Variations were observed based on the housing type (apartment blocks vs. single-story homes), physical ability (especially grip strength and balance), and availability of social support (e.g., help from neighbors or family). In multi-level dwellings without elevators, participants often delayed or minimized the waste disposal frequency due to the physical burden. Some developed compensatory strategies, such as accumulating waste near the door or using smaller, lighter bags to reduce the load.

Although a relatively brief activity, taking out the trash proved to be physically demanding. Four participants (SETs 6, 12, 14, and 21) were observed collecting waste from different rooms and disposing of it in outdoor or shared containers.

Key elements:

- Organized routine: Trash was consolidated before leaving the home, bags were tied securely, and containers were immediately replaced—demonstrating attention to hygiene and routine.
- Physical demands and movement: Tasks included lifting, navigating stairs or ramps, and walking long distances (up to 90 m). Barriers included weather conditions and irregular outdoor surfaces.
- Environmental usability: Participants often used carts, bags with handles, or improvised tools to reduce physical effort—highlighting mismatches between physical demands and available product design.
- Emotional dimension: Successfully completing this task provided a sense of order, pride, and satisfaction. Some participants also sorted recyclables or planned efficient disposal trips.

### 3.4. Cross-Activity Synthesis: Patterns and Contrasts

Across all three domestic tasks—cooking, dishwashing, and waste disposal—participants demonstrated a consistent awareness of their physical limitations and an active engagement in adapting their behavior accordingly. Despite variations in context, space, and physical ability, the data revealed a shared capacity for functional adaptation and situated problem-solving. These strategies were not isolated but often combined and adjusted dynamically throughout the day. Four main patterns were identified:

- Time management: Tasks were strategically distributed throughout the day in alignment with energy levels and pain cycles. For example, one participant cooked in the morning when standing was more tolerable, and they postponed dishwashing until the late afternoon. Similarly, waste disposal was often delayed until other errands justified leaving the house.
- Spatial adaptation: Participants adjusted their use of space to enhance comfort or compensate for environmental limitations. In kitchens with limited counter space, some repurposed nearby tables for food preparation. In homes with small sinks, participants used washbasins to rinse dishes or rearranged dish-drying areas. One participant with poor lighting in the dishwashing area relocated the task to a better-lit part of the home.
- Task delegation: When tasks exceeded physical capacity—such as lifting heavy waste bags or scrubbing pots—participants often relied on neighbors, family members, or domestic helpers. This delegation was typically framed not as dependency but as a rational allocation of effort.
- Improvised solutions: Many participants employed creative, low-cost adaptations to ease task demands. Examples include using kitchen tongs to handle hot lids, storing cleaning supplies in mobile containers, or placing cushions on chairs to reduce the strain during prolonged preparation tasks.

These adaptive strategies are often intersected with the contextual difference described earlier—such as manual ignition of water heaters or the need to navigate narrow, cluttered spaces—highlighting the complex interplay between the routine, environment, and user agency. The capacity for adaptation was not simply a matter of individual resilience, but a dynamic interaction between body, space, and habitus.

These adaptations were directly shaped by the usability, maintenance, and accessibility of household products. While many strategies were shared across tasks, each activity presented unique dynamics:

- Waste disposal: Marked by environmental barriers and physical demands.
- Dishwashing: Characterized by posture control, fatigue management, and symbolic notions of order and cleanliness.
- Cooking: Closely tied to cultural identity, care roles, and emotional fulfillment

These strategies reflect a significant capacity for problem-solving and self-management and can be interpreted as a form of emergent user solutions—functional innovations generated by users in response to real and situated challenge [44,45,46]. Such solutions often emerge without formal training or technical tools yet offer viable insights for inclusive product design.

On average, the observed tasks lasted between 25 and 50 min depending on the activity and physical condition of the participant. Cooking involved the most extended duration (often segmented throughout the day), while waste disposal was the shortest but often the most physically demanding. Several participants reported modifying the timing of these tasks to avoid fatigue, indicating a self-regulated balance between task complexity and perceived energy cost.

Table 2 synthesizes the observed functional adaptation strategies across the three key domestic tasks, organized by adaptation type. Each strategy reflects a pragmatic response to the physical and environmental constraints experienced by older adults and often involves simple, creative adjustments. Notably, many of these adaptations can be interpreted as emergent user solutions—user-generated innovations that arise without formal intervention but offer clear directions for inclusive product redesign [44]. The design insights proposed in the final column exemplify how these real-life strategies can inform the development of everyday tools that support aging in place.

These findings illustrate the everyday ingenuity and adaptability of older adults and provide a strong empirical basis for generating user-informed design criteria tailored to common domestic challenges.

## 4. Discussion

This study examined how functionally independent older adults in urban Chile adapt three everyday domestic tasks—cooking, dishwashing, and waste disposal—within their own homes. Contrary to initial expectations, the findings revealed that physical modifications of products were relatively uncommon. Instead, participants predominantly engaged in temporal, spatial, and behavioral adaptations to manage functional challenges. This suggests that aging in place is often sustained not through the transformation of objects, but through flexible and situated strategies embedded in daily routines.

In this study, we define emergent user solutions as informal, context-driven strategies developed by older adults to compensate for functional limitations or environmental barriers in everyday life. These responses are not pre-planned or externally provided, but arise spontaneously from users’ lived experience, accumulated expertise, and creative problem-solving. Such solutions can include object repurposing, spatial reconfiguration, pacing strategies, or informal assistance. This concept aligns with theories of situated action and adaptive behavior in gerontology [47], as well as user innovation frameworks in design [48], and underscores the active role of older adults as agents of functional adaptation rather than passive recipients of care. While some of the prior literature emphasizes the potential of redesigning everyday products as a means to support autonomy in older adults [15,26], our data indicate that few participants had altered the form or function of domestic objects in a sustained or material way. Only occasional examples were observed—such as wrapping cloth around utensil handles for a better grip, or stabilizing containers with surrounding objects—and these tended to be improvised, temporary, and resource-dependent. Their rarity may be explained by the material, technical, or social demands involved in such modifications, which often exceed the capacity or motivation of users operating in low-resource or informal settings.

Instead, participants displayed a high degree of pragmatic adaptability, using strategies that required fewer resources and could be adjusted dynamically. These included shifting tasks to different times of day to align with energy levels; relocating activities to more accessible or better-lit areas; coordinating with household members or neighbors to share or delegate physically demanding tasks; and repurposing spaces or objects in creative but non-permanent ways. These behaviors reflect what has been described as situated autonomy [17]: a form of independence that is actively negotiated through interaction with the environment, rather than guaranteed by static infrastructure or devices.

From a design perspective, this suggests that interventions to support aging in place should focus not only on the development of assistive products or home modifications but also on enhancing the flexibility, modularity, and accessibility of everyday domestic environments. Rather than assuming that older adults will modify products themselves, designers could focus on creating tools that are adaptable without alteration, and that better accommodate varying postures, energy levels, or workflows. Additionally, participatory and observational design methods can help uncover these subtle forms of adaptation that often go unnoticed in traditional needs assessments.

**Figure 6:**
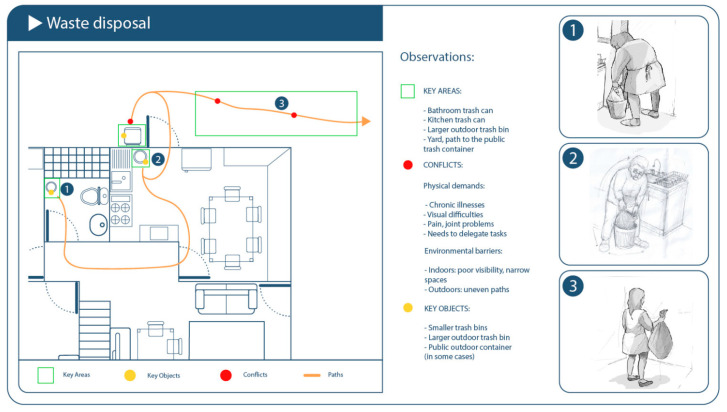
Schematic of the domestic environment observed during the waste disposal activity.

**Figure 7:**
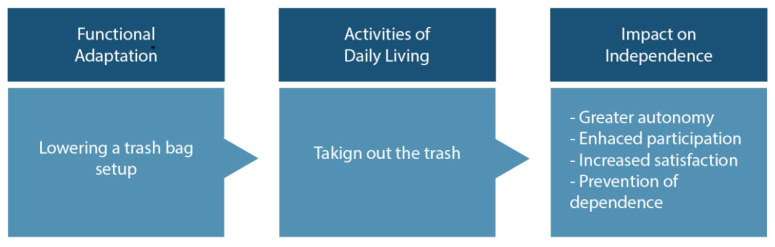
Conceptual diagram illustrates how a functional adaptation strategy (e.g., using a cart to transport garbage) in the task of “taking out the trash” supports autonomy (by avoiding physical overexertion), participation (by enabling routine waste disposal), and satisfaction (through successful task completion) and prevents dependency (by delaying the need for caregiver assistance).

Finally, the findings call for a broader understanding of what counts as adaptation in later life. Rather than prioritizing visible or engineered solutions, research and design practice must also value the silent, improvised, and socially embedded strategies through which older adults maintain autonomy on their own terms. For instance, adaptation in the task of “taking out the trash”, illustrated in Figure 6 and Figure 7, supports autonomy (by avoiding physical overexertion), participation (by enabling routine waste disposal), satisfaction (through successful task completion), and prevention of dependency (by delaying the need for caregiver assistance).

### 4.1. Implications

The findings of this study suggest at least three key areas for action, each of which can be operationalized through specific interventions and cross-sector collaboration.

#### 4.1.1. Product Design

There is a need for design approaches that prioritize modularity, ergonomics, ease of maintenance, and adaptability in everyday domestic tools. Inclusive product design frameworks should accommodate the capabilities and preferences of older adults while also recognizing the diversity of strategies they employ to address daily challenges.

Instead of designing from assumptions, it is possible to construct viable and situated iterations based on user-generated adaptations. For example, kitchen utensils can be redesigned with thicker, non-slip handles to accommodate a reduced grip strength, and dishwashing stations can incorporate height-adjustable surfaces or fold-out seating to reduce postural strain. These directions align with the User-Driven Minimum Feasible Product model [49] and can be directly translated into inclusive design briefs or student co-design workshops focused on aging. This approach resonates with established principles of inclusive and age-aware design, which emphasize physical accessibility, cognitive clarity, and adaptability across user profiles [50].

#### 4.1.2. Functional Health Promotion

Community health programs can integrate training in ergonomic techniques, promote adaptive strategies, and encourage the use of simple assistive tools that are already present in homes. This could include local workshops that teach older adults how to pace household tasks, reuse everyday objects (e.g., stools, carts) for ergonomic support, or reorganize spaces to reduce effort and improve safety.

Such interventions can be co-developed with primary care teams and implemented in partnership with senior centers, allowing health promotion to extend beyond clinical care into the domestic environment where autonomy is negotiated daily.

In addition to supporting autonomy, many of the observed adaptive strategies serve to mitigate environmental risks. For instance, organizing kitchen tasks to minimize reaching or bending, or using carts to transport waste, directly address the biomechanical vulnerabilities of aging bodies. These adaptations are especially critical given the high incidence of falls and injuries during home-based tasks. Data from the World Health Organization indicate that falls are the second-leading cause of accidental injury deaths worldwide, disproportionately affecting people aged 65 and older [51]. Designing with these realities in mind can transform ordinary routines into safer, more sustainable actions.

#### 4.1.3. Public Policy

Policies related to aging must move beyond a focus on architectural infrastructure and include improvements in the usability and accessibility of everyday products. Specific policy actions could include micro-grants for ergonomic household modifications, municipal waste collection services designed with senior-friendly features (e.g., smaller containers, community drop points), and national standards for the inclusive design of common kitchen tools.

Supporting inclusive and affordable innovation in product design can reduce the risk of loss of autonomy and help lower long-term healthcare costs. Moreover, public procurement mechanisms could favor products that demonstrate adherence to ergonomic and aging-in-place principles, thus bridging the gap between research insights and market transformation.

### 4.2. Limitations and Future Research

This study is limited by its qualitative scope and small sample size. While it enabled an in-depth exploration of lived experiences, the findings are not generalizable to broader populations. Additionally, participants’ behavior may have been influenced by the presence of the observer—a phenomenon known as the Hawthorne effect—which could have altered routine behaviors in subtle ways.

Another limitation lies in the absence of formal functional or cognitive assessments (e.g., ADL/IADL scales or physical capacity tests), which limits the comparability across levels of independence. This study also focused on urban older adults in southern Chile, leaving open questions about cross-regional and cultural differences.

Future research could explore the following directions:

- Integrating mixed methods that combine qualitative data with functional assessments or sensor-based monitoring.
- Conducting longitudinal studies to understand how adaptive strategies evolve over time and across different stages of aging.
- Comparing urban and rural environments, or including older adults with varying levels of dependency, to capture broader contextual variation.
- Developing and validating assessment tools that measure functionality, usability, and maintenance of household objects from both gerontological and design perspectives.
- Exploring the relationship between task duration, perceived effort, and autonomy, to better inform design and care strategies that reduce energy costs without undermining participation.

## 5. Conclusions

This qualitative study explored in depth how functionally independent older adults carry out three key domestic activities—cooking, dishwashing, and waste disposal—in urban areas of southern Chile. Through situated observation and inductive analysis, we identified patterns of functional adaptation and compensatory strategies that reflect a dynamic interplay between the body, household objects, and the built environment.

The findings demonstrate that autonomy in later life is not solely determined by the absence of illness or physical decline. Instead, it strongly depends on the ability of older adults to reorganize, adapt, and re-signify their interactions with domestic routines and objects. In all the activities analyzed, we observed persistent tensions between the physical and symbolic demands of the task and the characteristics of the tools and settings involved.

These tensions highlight a continuous gap between actual functional needs and the current design of household products. This underscores the urgency of incorporating usability, ergonomic, and accessibility criteria not only into specialized assistive technologies but also into everyday objects. Furthermore, the strategies observed suggest that low-cost, high-impact innovations are possible when the situated knowledge and adaptive behaviors of older adults are treated as meaningful design inputs.

From a broader perspective, our findings support the view that domestic objects and environments should be considered integral components of health promotion and care systems for aging populations. Integrating user-centered design for older adults into public policy, community rehabilitation programs, and technological development can significantly contribute to extending functional autonomy, improving quality of life, and reducing both the economic and emotional costs of aging.

This study provides clear evidence that older adults maintain functional autonomy not despite ergonomic limitations or environmental barriers, but through creative and flexible adaptation strategies. Observing and understanding these strategies offers a valuable path for innovation in product design, public health, and aging policy—centered not only on needs but also on capabilities.

These insights may also inform future participatory and situated design methodologies that start from user-generated adaptations rather than idealized models of product use.

## Figures and Tables

**Table 1:** Exemplar participant quotes.

Theme	Description	Exemplar Quote
Temporal Redistribution	Adjusting the timing or pacing of tasks to match energy levels or reduce strain.	“I cook in the morning when I feel stronger; in the evening I just heat things up”.
Ergonomic Adaptation	Modifying the posture or workspace or using supports to reduce physical effort.	“I keep a stool near the sink so I can sit while doing the dishes”.
Improvised Solutions	Using household objects in creative or unconventional ways to solve problems.	“I use a plastic bottle as a funnel because my hands don’t grip well anymore”.
Symbolic Meaning of Routines	Performing tasks that reinforce identity, autonomy, or personal satisfaction.	“Cleaning the kitchen makes me feel good, like everything is in its place again”.

**Table 2:** Functional adaptation strategies observed across domestic activities and their potential design insights.

Activity	Adaptation Type	Strategy	Design Insight/Possible Solution
Cooking	Temporary	Using a stool while preparing meals	Adjustable-height stools with ergonomic back support and anti-slip bases
	Spatial	Locating all utensils within reach	Modular storage systems with easy-access drawers for essential items
	Technological	Using electric kettles or microwaves to preheat water or soften food, reducing manual effort	Design intuitive, low-effort devices with large buttons, visible indicators, and automatic shut-off for safety and ease of use
	Social	Seeking help from a family member	Visual instructions on appliance use to support intergenerational assistance
Washing	Temporary	Seated bathing	Foldable bath stools with drainage design and anti-tip structure
	Spatial	Moving the washing machine closer	Compact, front-loading machines with wall-mounted controls
	Technological	Handheld shower head	Ergonomic, pressure-adjustable showerheads with magnetic holders
	Social	Seeking help from a family member	Labels and flowcharts for simple task sharing in shared spaces
Taking Out the Trash	Temporary	Placing the bag on a chair	Wheeled trash bag support stands with adjustable height
	Spatial	Placing the bin near the door	Indoor-to-outdoor transition carts for trash, weather-resistant
	Technological	Light garbage bags	Biodegradable, ultra-light bags with reinforced handles
	Social	Asking a neighbor to take it out	Community noticeboards or digital alerts to coordinate neighbor support

## Data Availability

The datasets used during the current study are available from the corresponding author on reasonable request.

## Supplementary Materials

The following supporting information can be downloaded at: https://www.mdpi.com/article/10.3390/healthcare13121369/s1.

## References

[1] WHO (2022). 10 Facts on Ageing and Health. https://www.who.int/news-room/fact-sheets/detail/ageing-and-health.

[2] Wiles J.L., Leibing A., Guberman N., Reeve J., Allen R.E.S. (2011). The Meaning of “Aging in Place” to Older People. Gerontologist.

[3] Smith R.J., Baik S., Lehning A.J., Mattocks N., Cheon J.H., Kim K. (2022). Residential Segregation, Social Cohesion, and Aging in Place: Health and Mental Health Inequities. Gerontologist.

[4] Edemekong P.F., Bomgaars D.L., Sukumaran S., Schoo C. (2023). Activities of Daily Living. StatPearls.

[5] Kötteritzsch A., Weyers B. (2016). Assistive Technologies for Older Adults in Urban Areas: A Literature Review. Cogn. Comput..

[6] Ma C., Guerra-Santin O., Mohammadi M. (2022). Smart Home Modification Design Strategies for Ageing in Place: A Systematic Review. J. Hous. Built Environ..

[7] Quesada-García S., Valero-Flores P., Lozano-Gómez M. (2023). Active and Assisted Living, a Practice for the Ageing Population and People with Cognitive Disabilities: An Architectural Perspective. Int. J. Environ. Res. Public Health.

[8] Berrett J., de Kruiff A., Pedell S., Reilly A. (2022). Augmented Assistive Technology: The Importance of Tailoring Technology Solutions for People Living with Dementia at Home. Int. J. Hum. Comput. Stud..

[9] Daban F., Garcia-Subirats I., Porthé V., López M.J., de-Eyto B., Pasarín M.I., Borrell C., Artazcoz L., Pérez A., Díez E. (2021). Improving Mental Health and Wellbeing in Elderly People Isolated at Home Due to Architectural Barriers: A Community Health Intervention. Aten. Primaria.

[10] Engineer A., Sternberg E.M., Najafi B. (2018). Designing Interiors to Mitigate Physical and Cognitive Deficits Related to Aging and to Promote Longevity in Older Adults: A Review. Gerontology.

[11] Andrew S., Gloria G. (2013). Technologies for Active Aging.

[12] Cicirelli G., Marani R., Petitti A., Milella A., D’Orazio T. (2021). Ambient Assisted Living: A Review of Technologies, Methodologies and Future Perspectives for Healthy Aging of Population. Sensors.

[13] Fallah H., Nazari J., Choobineh A., Morowatisharifabad M.A., Jafarabadi M.A. (2021). Identifying Barriers and Problems of Physical Environment in Older Adults’ Homes: An Ergonomic Approach. Work.

[14] Crews D.E. (2022). Aging, Frailty, and Design of Built Environments. J. Physiol. Anthropol..

[15] Kuoppamäki S., Tuncer S., Eriksson S., McMillan D. (2021). Designing Kitchen Technologies for Ageing in Place. Proc. ACM Interact. Mob. Wearable Ubiquitous Technol..

[16] Chavarria M.A., Schönenberger K., Mugeere A., Hurst S., Velarde M.R. (2021). Design Approaches for Creating Person-Centered, Context Sensitive, and Sustainable Assistive Technology with the Global South. Accessible Technology and the Developing World.

[17] Ballmer T., Gantschnig B. (2024). Maintaining Autonomy: How Older Persons with Chronic Conditions and Their Significant Others Interpret, Navigate, and Overcome Everyday Difficulties. Scand. J. Occup. Ther..

[18] Kelly A.J., Fausset C.B., Rogers W., Fisk A.D. (2012). Responding to Home Maintenance Challenge Scenarios. J. Appl. Gerontol..

[19] Arribas Velasco A., McGrory J., Berry D. (2023). An Evaluation Study on the Analysis of People’s Domestic Routines Based on Spatial, Temporal and Sequential Aspects. Appl. Sci..

[20] Boffi M., Pola L., Fumagalli N., Fermani E., Senes G., Inghilleri P. (2021). Nature Experiences of Older People for Active Ageing: An Interdisciplinary Approach to the Co-Design of Community Gardens. Front. Psychol..

[21] Miśniakiewicz A. (2024). Co-Creating a Seniors’ Meeting Place: A Prototype Pop-Up Installation on a Popowice Housing Estate in Wrocław, Poland. Buildings.

[22] Fumagalli N., Fermani E., Senes G., Boffi M., Pola L., Inghilleri P. (2020). Sustainable Co-Design with Older People: The Case of a Public Restorative Garden in Milan (Italy). Sustainability.

[23] Curumsing M.K., Fernando N., Abdelrazek M., Vasa R., Mouzakis K., Grundy J. (2019). Emotion-Oriented Requirements Engineering: A Case Study in Developing a Smart Home System for the Elderly. J. Syst. Softw..

[24] Probst F., Ratcliffe J., Molteni E., Mexia N., Rees J., Matcham F., Antonelli M., Tinker A., Shi Y., Ourselin S. (2024). A Scoping Review on Human-Centered Design Approaches and Considerations in the Design of Technologies for Loneliness and Social Isolation in Older Adults. Des. Sci..

[25] Lawton M.P., Nahemow L., Eisdorfer C., Lawton M.P. (1973). Ecology and the aging process. The Psychology of Adult Development and Aging.

[26] Zajicek M. (2005). Older Adults: Key Factors in Design. Future Interaction Design.

[27] Langley J., Wheeler G., Partridge R., Bec R., Wolstenholme D., Sproson L. (2020). Designing with and for Older People. Designing with and for Older People.

[28] Roos V., van Rensburg J.T.J. (2022). Inclusion of Marginalized Older Individuals in Artefact Design: Reflections and Recommendations. Age-Inclusive ICT Innovation for Service Delivery in South Africa.

[29] Elliott N., Lazenbatt A. (2005). How to Recognise a “Quality” Grounded Theory Research Study. Aust. J. Adv. Nurs..

[30] Rai N., Thapa B. (2015). A Study on Purposive Sampling Method in Research. Kathmandu Sch. Law.

[31] Smith J., Charmaz (2015). Grounded Theory. Qualitative Psychology: A Practical Guide to Research Methods.

[32] Vasileiou K., Barnett J., Thorpe S., Young T. (2018). Characterising and justifying sample size sufficiency in interview-based studies: Systematic analysis of qualitative health research over a 15-year period. BMC Med. Res. Methodol..

[33] Hladkowicz E., Auais M., Kidd G., McIsaac D.I., Miller J. (2024). “It’sa stressful, trying time for the caretaker”: An interpretive description qualitative study of postoperative transitions in care for older adults with frailty from the perspectives of informal caregivers. BMC Geriatr..

[34] Pedersen A.K., Ernstsen L. (2025). Instrumental activities of daily living and self-rated health in community-dwelling older adults: Cross-sectional findings from the HUNT Study (HUNT4 Trondheim 70+). BMC Geriatr..

[35] Ibrahim N.I., Davies S. (2012). Aging: Physical difficulties and safety in cooking tasks. Work.

[36] Lee M., Mishra R.K., Momin A., El-Refaei N., Bagheri A.B., York M.K., Najafi B. (2022). Smart-home concept for remote monitoring of instrumental activities of daily living (IADL) in older adults with cognitive impairment: A proof of concept and feasibility study. Sensors.

[37] Simons H., Leavy P. (2014). Case Study Research: In-Depth Understanding in Context. The Oxford Handbook of Qualitative Research.

[38] Oktay J.S. (2012). Grounded Theory.

[39] Spinellis D. (2003). Code Reading: The Open-Source Perspective.

[40] Hwang S. (2008). Utilizing Qualitative Data Analysis Software. Soc. Sci. Comput. Rev..

[41] Nowell L.S., Norris J.M., White D.E., Moules N.J. (2017). Thematic analysis: Striving to meet the trustworthiness criteria. Int. J. Qual. Methods.

[42] McCambridge J., Witton J., Elbourne D.R. (2014). Systematic review of the Hawthorne effect: New concepts are needed to study research participation effects. J. Clin. Epidemiol..

[43] Tong A., Sainsbury P., Craig J. (2007). Consolidated criteria for reporting qualitative research (COREQ): A 32-item checklist for interviews and focus groups. Int. J. Qual. Health Care.

[44] Stegner L., Senft E., Mutlu B. (2023). Situated Participatory Design: A Method for In Situ Design of Robotic Interaction with Older Adults. Proc. CHI Conf. Hum. Factors Comput. Syst..

[45] De Moor K., Berte K., De Marez L., Joseph W., Deryckere T., Martens L. (2010). User-Driven Innovation? Challenges of User Involvement in Future Technology Analysis. Sci. Public Policy.

[46] Peters J., Bleakney A., Sornson A., Hsiao-Wecksler E., McDonagh D. (2024). User-Driven Product Development: Designed by, Not Designed for. Des. J..

[47] Clark F., Carlson M., Zemke R., Frank G., Patterson K., Ennevor B.L., Lipson L. (1996). Life domains and adaptive strategies of a group of low-income, well older adults. Am. J. Occup. Ther..

[48] von Hippel E. (2005). Democratizing Innovation.

[49] Nicklas S.J., Atzberger A., Briede-Westermeyer J.C., Paetzold K. (2020). The User-Driven Minimum Feasible Product–Towards a Novel Approach on User Integration. Proc. Des. Soc. Des. Conf..

[50] Clarkson P.J., Coleman R., Keates S., Lebbon C. (2003). Inclusive Design: Design for the Whole Population.

[51] World Health Organization (2023). Falls. https://www.who.int/news-room/fact-sheets/detail/falls.

