# Supplementary material for: Functional Adaptation and Emergent User Solutions in Domestic Tasks: Supporting Aging in Place Through a Field Study on Design Challenges Among Older Adults in Chile
Source: Healthcare (Basel). 2025 Jun 7;13(12):1369. doi: 10.3390/healthcare13121369 (PMC12192774; doi:10.3390/healthcare13121369)
Supplement: Supplementary file 1 [file healthcare-13-01369-s001.zip › healthcare-3642755-supplementary.pdf]

Criteria for Reporting Qualitative Research

(Tong, A., Sainsbury, P., & Craig, J., 2007)

Domain 1: Research team and reflexivity

Personal characteristics

1. **Interviewer/facilitator** – The main data collector (first author) conducted observations and interviews.
2. **Credentials** – Academic and professional credentials of the authors are noted in the author affiliations.
3. **Occupation** – Researchers are affiliated with design and health faculties, actively engaged in aging and design research.
4. **Gender** – The gender of field researchers (male and female) is implicitly acknowledged and balanced.
5. **Experience and training** – The research team had prior experience with qualitative methods and work with older adults.

Relationship with participants

6. **Relationship established** – Participants were contacted via community networks; some rapport was established before observation.
7. **Participant knowledge of the interviewer** – Participants were informed of the purpose of the study and the researcher's role.
8. **Interviewer characteristics** – Reflexivity regarding the researchers' design/health background is addressed in the methodology section.

Domain 2: Study design

Theoretical framework

9. **Methodological orientation** – The study followed grounded theory principles and thematic analysis, as stated in Methods.

Participant selection

10. **Sampling** – Purposeful sampling was used, based on a previous large-scale survey.
11. **Method of approach** – Participants were approached via local community organizations and in-person visits.
12. **Sample size** – Twenty participants were included, justified by theoretical saturation.
13. **Non-participation** – No dropouts were reported; all invited participants consented and completed the process.

Setting

14. **Setting of data collection** – Observations took place in participants' homes during real tasks.
15. **Presence of non-participants** – Occasionally family members were present, noted in

the field reports.
16. **Description of sample** – Demographic characteristics (age, gender, living arrangement, SES) are detailed in Methods.

Data collection
17. **Interview guide** – A structured observation protocol and guiding questions were used.
18. **Repeat interviews** – Not conducted; data was collected through single-session observations or interviews.
19. **Audio/visual recording** – Field notes and photographs were used; no audio recording due to the observational nature.
20. **Field notes** – Extensive field notes were recorded during and after observations.
21. **Duration** – Sessions varied between 30 minutes and 2 hours depending on activity.
22. **Data saturation** – Discussed in relation to the grounded theory approach (achieved by 18th participant).
23. **Transcripts returned** – Transcripts were not returned; however, interpretations were triangulated among researchers.

🟩 Domain 3: Analysis and findings

Data analysis
24. **Number of data coders** – At least two coders analyzed the data collaboratively.
25. **Description of the coding tree** – A visual coding tree (Figure S1) was provided.
26. **Derivation of themes** – Themes emerged inductively from the data using grounded theory.
27. **Software** – ATLAS.ti v23 was used for qualitative data analysis.
28. **Participant checking** – Member checking was not conducted; however, triangulation and consistency checks were used.

Reporting
29. **Quotations presented** – Representative participant quotes are presented in Table 2.
30. **Data and findings consistent** – Findings are well-supported by data and direct observations.
31. **Clarity of major themes** – Major themes are clearly stated and discussed in Results and Discussion.
32. **Clarity of minor themes** – Variations and contextual nuances are reported and interpreted.
